# 658. Evolving Infection Patterns and Mortality Risk in Hypervirulent Klebsiella pneumoniae Bloodstream Infections: A 10-Year Single-Center Study

**DOI:** 10.1093/ofid/ofaf695.213

**Published:** 2026-01-11

**Authors:** Keon Young Lee, Seong Hun Jeong, Miri Hyun, Ji Yeon Lee, Hyun ah Kim

**Affiliations:** Keimyung University Dongsan Hospital, Keimyung University School of Medicine, Daegu, Taegu-jikhalsi, Republic of Korea; Keimyung University Dongsan Hospital, Keimyung University School of Medicine, Daegu, Taegu-jikhalsi, Republic of Korea; Keimyung University Dongsan Hospital, Keimyung University School of Medicine, Daegu, Taegu-jikhalsi, Republic of Korea; Keimyung University Dongsan Hospital, Keimyung University School of Medicine, Daegu, Taegu-jikhalsi, Republic of Korea; Department of Internal Medicine, Keimyung University Dongsan Medical Center, Daegu, Korea, Daegu, Taegu-jikhalsi, Republic of Korea

## Abstract

**Background:**

Hypervirulent *Klebsiella pneumoniae* (hvKP) bloodstream infections (BSIs) have traditionally been associated with favorable outcomes. However, with increasing emergence of antimicrobial resistance, clinical impact of hvKP is evolving. We aimed to identify clinical and microbiological factors, including virulence factors, associated with 30-day mortality in hvKP BSIs.

**Figure:**
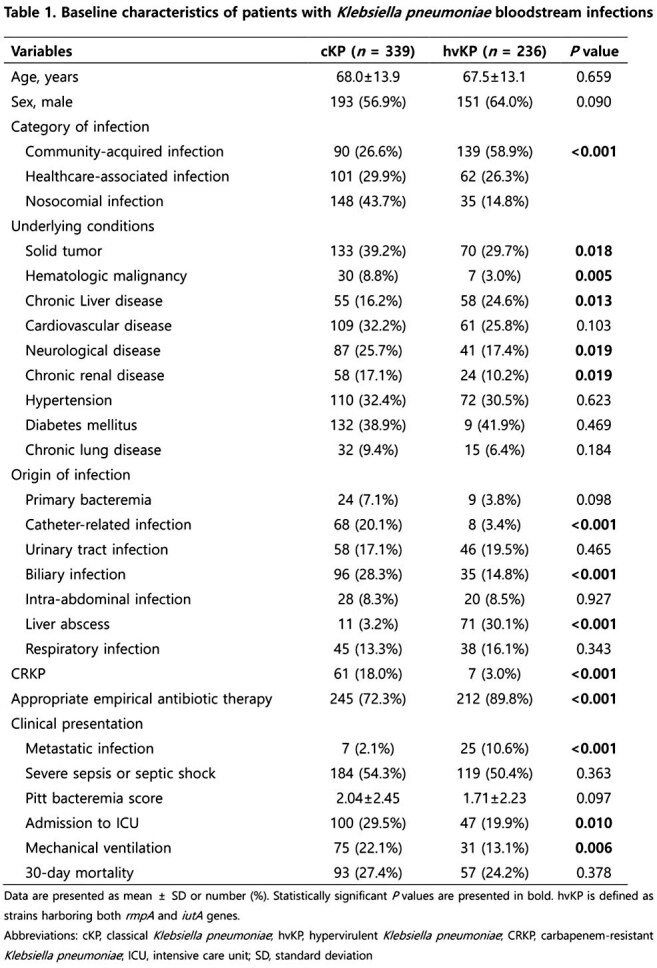

**Figure:**
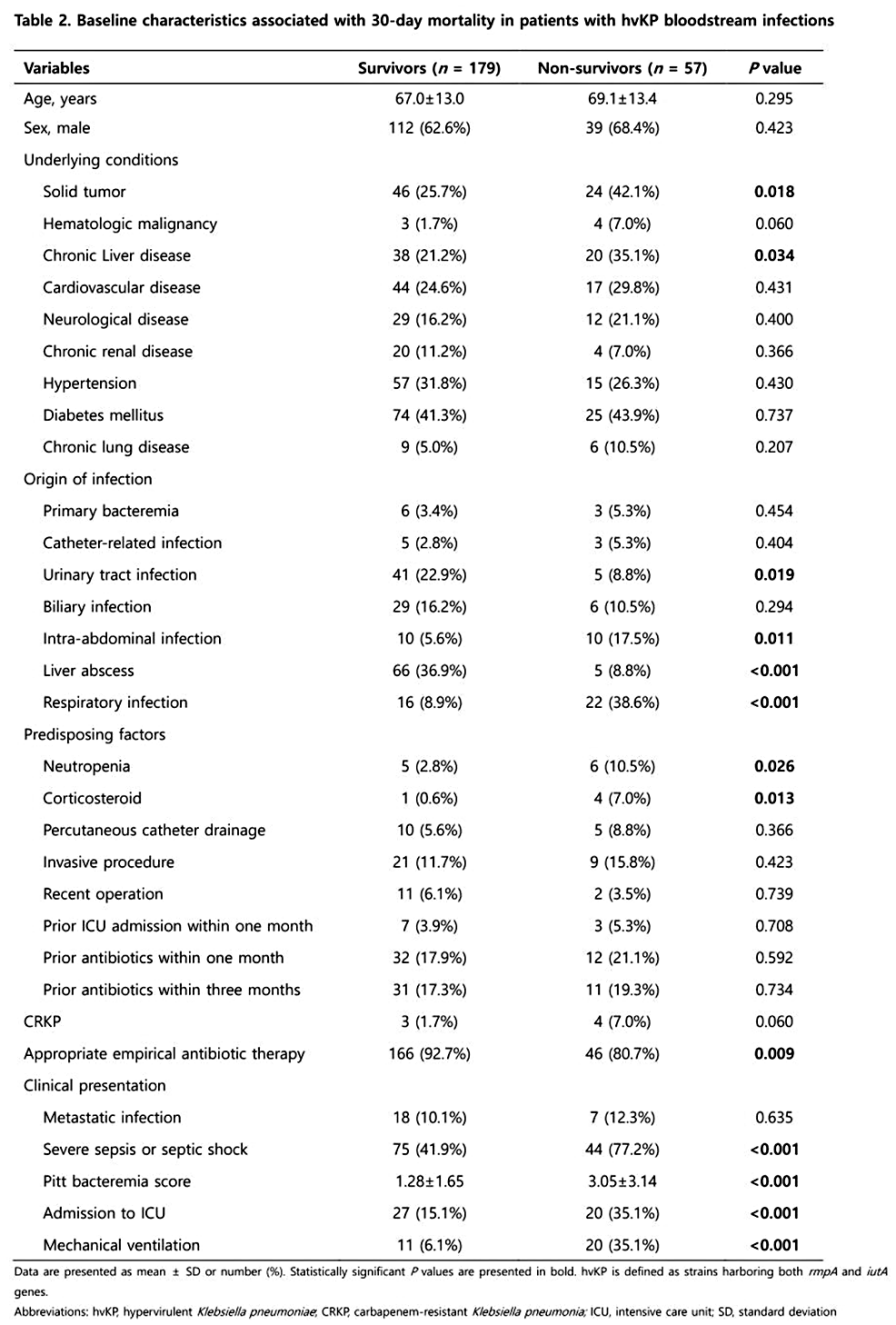

**Methods:**

We retrospectively analyzed 575 patients with *Klebsiella pneumoniae* (KP) BSIs at Keimyung University Dongsan Hospital from January 2014 to December 2023. hvKP was defined by the presence of both *rmpA* and *iutA* genes. Clinical characteristics, antimicrobial resistance, virulence factors were compared between the survivors and non-survivors based on 30-day mortality outcomes. Multivariate logistic regression was used to identify independent risk factors for 30-day mortality.

**Figure:**
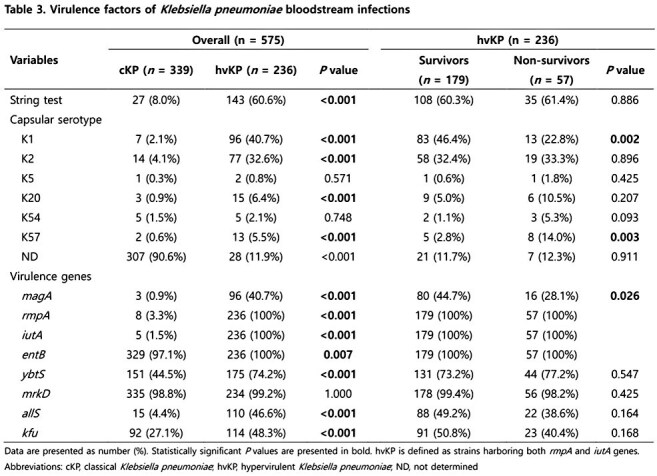

**Figure:**
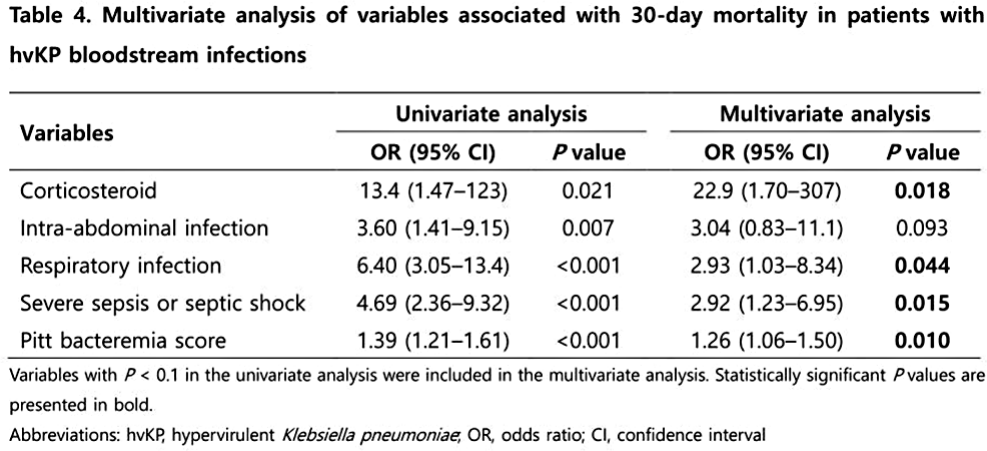

**Results:**

Among 575 KP BSIs, 236 (41.0%) were identified as hvKP. In hvKP BSIs, while liver abscess was the predominant source, a notable proportion arose from urinary tract and respiratory infections (30.1% vs. 19.5%, 16.1%). Among hvKP patients, respiratory infection was strongly associated with poor outcomes, with a 30-day mortality of approximately 57.8%. Antimicrobial resistance rates, including carbapenem resistance, did not significantly differ between survivors and non-survivors, though carbapenem-resistant hvKP (3.0%) was noted. K1 serotype and *magA* gene were associated with survival, whereas K57 serotype was linked to higher mortality. In multivariate analysis, respiratory infection, septic shock, corticosteroid use, and higher Pitt bacteremia score were identified as independent risk factors for 30-day mortality.

**Conclusion:**

hvKP BSIs do not necessarily result in better outcomes compared to classical KP. Respiratory infection emerged as a key risk factor for mortality, emphasizing the need for aggressive early management. While antimicrobial resistance was not directly associated with mortality, the emergence of carbapenem-resistant hvKP warrants attention. Capsular serotype analysis revealed that K1 correlated with better outcomes, while K57 was linked to worse prognosis. hvKP BSIs require vigilant management due to their severe clinical presentations and emerging antimicrobial resistance.

**Disclosures:**

All Authors: No reported disclosures

